# On the Subcutaneous Injection of Blood, Infusion (Subcutaneous) and Intravenous Transfusion

**Published:** 1888-01

**Authors:** Frank A. Harrington

**Affiliations:** Assistant to the Chair of Materia Medica, Niagara University


### Translations.

### *ON THE SUBCUTANEOUS INJECTION OF BLOOD, INFUSION (SUBCUTANEOUS) AND INTRAVENOUS TRANSFUSION.*

A Translation of an Abstract by Oehler, in the *Centralblatt für Chirurgie.* of Ziemssen's paper on this subject.

By FRANK A. HARRINGTON, M. D., Assistant to the Chair of Materia Medica, Niagara University.

Ziemssen states, in the first place, that it is nonsense to discuss intravenous transfusion : it is attended by so many dangerous phenomena, such as chills, fevers, albuminuria, hæmoglobinuria, etc. ; instead, it should be considered an operation directly endangering life. However, this unfortunate result is not dependent on the introduction of defibrinated blood, but in the manner of performing intravenous injection, by which altered blood, containing ferments, unavoidable flocculi of fibrin and air-bubbles, are injected into the vessels. It is proven that all dangers are obviated if the blood be injected into the subcutaneous cellular tissue. Ziemssen's process is : blood, completely defibrinated, which must be carefully used with aseptic precautions, and preserved at a temperature between 37° and 40°, is injected, by means of a syringe containing twenty-five cubic centimeters, into the subcutaneous tissue, preferentially into the thigh, and forcibly rubbed by an assistant. This massage is a very necessary step in the procedure. When a larger amount is to be introduced, Ziemssen claims it is absolutely necessary that the patient be anæsthetized : the operation is attended with so much pain ; that is an objection to the process. Each syringeful is injected into a different place. Very large amounts can be injected, and Ziemssen has used as much as 350 grammes, introducing it at fourteen distinct places. To mitigate any pain succeeding the operation, an icebag is applied, and the patient kept quiet. In the first place, the affair is unaccompanied by danger. Only in two cases was any suppuration noticed, and that, a consequence of trifling mistakes that were rectifiable. No fever, no chill, no albuminuria, no symptoms of hæmoglobinuria succeeded. The subcutaneous cellular tissue serves as an excellent filter to retain the ferments and air-bubbles, while the red corpuscles easily and quickly pass through. As evidence of this, you find, after a few days, no trace of free blood, while, in the circulation, the hæmoglobin is shown to be increased. In the first twenty-four hours, this increases in many cases twofold. To be sure, this result is not a certainty ; oftener, during the ensuing four or five days, there is a slow decrease of hæmoglobin down to a stationary amount, which is, nevertheless, always greater than the original amount before any injection. By a repetition of the operation many times, the hæmoglobin increases more and more, until ultimately it reaches the normal standard. Hand in hand with that, there goes on an evident increase in the number of red corpuscles. Ziemssen, in this way, succeeded in curing, in the course of a month, various forms of chronic anæmia of a bad type. In cases of acute anæmia, after a large loss of blood, Ziemssen has not tested his method ; still he believes the subcutaneous injection of blood is indicated in such cases. Of course, when delay is dangerous, and when, for some reason, this injection of blood cannot be made, Ziemssen employs the subcutaneous injection of salt and water. This, again, has the advantage over intravenous injection, of being easier performed, and wholly without danger. It is not necessary to use distilled water; any spring water, provided it has been well sterilized by boiling, can be unhesitatingly injected. The subcutaneous cellular tissue retains any foreign solid matter after it has been made aseptic. The water is so easily absorbed, that anæsthesia can be dispensed with. To introduce 600 grammes of water, one needs to make four or five punctures. If, in the injection of water, the danger is met with dependent on an "empty heart," then should the injection of blood be quickly prepared ; for, as experience teaches, the injection of salt and water merely delays, not prevents death. In such a case, fresh blood must be introduced into the body. We hope these methods of transfusion will also be useful in acute anæmia.

At all events, the small pamphlet by Ziemssen, written in so clear and masterly a manner, offers much of clinical interest in the important subject of transfusion.

